# The Polyscore of autonomic parameters predicts mortality and identifies low-risk individuals among diabetic survivors of acute myocardial infarction

**DOI:** 10.1038/s41598-022-09899-y

**Published:** 2022-04-12

**Authors:** Alexander Steger, Michael Dommasch, Alexander Müller, Petra Barthel, Daniel Sinnecker, Larissa Wieg, Alexander Hapfelmeier, Helene Hildegard Heidegger, Katharina Maria Huster, Eimo Martens, Karl-Ludwig Laugwitz, Georg Schmidt, Ralf Dirschinger

**Affiliations:** 1 Klinik und Poliklinik für Innere Medizin I, University Hospital, Technical University of Munich, Ismaninger Straße 22, 81675 Munich, Germany; 2 DZHK (German Centre for Cardiovascular Research), Partner Site Munich Heart Alliance, Munich, Germany; 3 MVZ Harz, Kösliner Straße12, 38640 Goslar, Germany; 4 School of Medicine, Institute for AI and Informatics in Medicine, Technical University of Munich, Ismaninger Straße 22, 81675 Munich, Germany; 5 School of Medicine, Institute of General Practice and Health Services Research, Technical University of Munich, Orleansstraße 47, 81667 Munich, Germany; 6 Department of Obstetrics and Gynecology, University Hospital, LMU Munich, Maistraße 11, 80337 Munich, Germany

**Keywords:** Predictive medicine, Prognostic markers, Myocardial infarction, Endocrine system and metabolic diseases, Prognosis, Risk factors, Outcomes research

## Abstract

Survivors of an acute myocardial infarction with diabetes mellitus retain an increased mortality risk. Reliable assessment of individual risk is required for effective and cost-efficient medical care in these patients. The Polyscore is a previously established risk predictor consisting of seven autonomic tests derived from electrocardiogram, blood pressure, and respiration. The Polyscore allows classification of survivors of myocardial infarction in groups at low, intermediate and high mortality risk. The aim of this study was to investigate the prognostic value of the Polyscore in diabetic survivors of acute myocardial infarction, which may be impaired by the presence of diabetic autonomic neuropathy. Survivors of an acute myocardial infarction were included in a prospective cohort study during hospitalisation due to the index event at two university hospitals in Munich, Germany. The Polyscore was determined from simultaneous non-invasive 30-min recordings of electrocardiogram, continuous arterial blood pressure, and respiration which were performed in all participants. Patients were followed for 5 years. The primary and secondary outcomes were all-cause mortality and cardiac mortality. 184 of 941 enrolled patients (19.6%) suffered from diabetes mellitus. 5-year-mortality was higher in diabetic patients (15.2%) compared to non-diabetic patients (5.8%). A multivariable Cox regression model confirmed the Polyscore as a strong predictor of mortality in diabetic post-MI patients (intermediate risk: HR 6.56, 95% CI 1.61–26.78, p = 0.004, mortality 22.8%; high risk: HR 18.76, 95% CI 4.35–80.98, p < 0.001, mortality 68.8%). There was no interaction between diabetes mellitus and the Polyscore regarding mortality prediction (p = 0.775). Interestingly, in contrast to the groups at intermediate and high risk (73 patients, 39.7%), the Polyscore identified a majority of diabetic patients (111, 60.3%) with a low mortality risk, comparable to that of low-risk non-diabetic patients (3.6% and 2.1%, respectively, p = 0.339). Consistent results were observed for cardiac mortality. This analysis shows that the Polyscore predicts all-cause and cardiac mortality in diabetic survivors of acute myocardial infarction. Within these patients it identifies a large population not affected by the excess mortality associated with diabetes in this setting. Thus, the Polyscore may facilitate risk-adapted follow-up strategies in diabetic survivors of myocardial infarction.

## Introduction

Diabetes mellitus is one of the leading causes of death in the developed world^1^. The coincidence with cardiovascular disease and cardiovascular death is high^2^. Diabetic patients who have survived an acute myocardial infarction are at a substantially higher mortality risk compared to non-diabetic patients^3,4^. Besides glucose control, a major aspect of an effective and cost-efficient medical care is the early and reliable risk stratification of these patients. For that purpose, the development and validation of non-invasive risk stratification strategies is of utmost importance, especially for those diabetic patients with coexisting cardiovascular conditions.

Since autonomic nervous control and reflexes maintain the homeostasis of the organism, it is not surprising that their characterization provides strong prognostic information, thus allowing individual risk assessment^5^. The Polyscore is a novel risk predictor consisting of seven predominantly autonomic tests. They can be easily derived from simultaneous non-invasive short-term (30 min) recordings of electrocardiogram, blood pressure, and respiration. The Polyscore was developed in a cohort of patients who survived an acute myocardial infarction (ART study) and it divided this cohort in a small group at substantially increased mortality risk from much larger groups at low or intermediate mortality risk, facilitating risk-adapted follow-up strategies^5,6^.

However, in diabetic survivors of acute myocardial infarction, a disturbed autonomic function may be either related to the myocardial infarction itself or to a preexisting diabetic autonomic neuropathy^7,8^. Consequently, risk prediction based on the assessment of autonomic parameters might be of reduced value in this patient group. Therefore, the aim of this subanalysis of the ART study was to investigate the prognostic performance of the Polyscore in the clinical context of acute myocardial infarction complicated by a diabetic metabolic state.

## Methods

### Population

The population of this study has been described previously^6^. Survivors of an acute myocardial infarction were enrolled between May 2000 and March 2005 at two large university hospitals, the Klinikum rechts der Isar and the German Heart Centre, both located in Munich, Germany. Patients were included in this study during the initial hospitalisation due to the index myocardial infarction if they met the following criteria: age ≤ 80 years, sinus rhythm, no secondary prophylaxis indication for an implantable cardioverter defibrillator. The study was approved by the ethics committee of the Technical University of Munich and informed consent was obtained from all participants. All research was performed in accordance with relevant guidelines/regulations and the study was conducted in accordance with the Declaration of Helsinki.

### Clinical data

An acute myocardial infarction was diagnosed, if two of the following criteria were present: typical chest pain, creatine kinase above twice the upper limit of normal, admission ST segment elevations that were diagnostic for myocardial infarction. Diabetes mellitus was considered present if a patient was already diagnosed and was receiving treatment (diet, tablets, and/or insulin) or if occasional plasma glucose levels repeatedly exceeded 11 mmol/L. The left ventricular ejection fraction was assessed either by echocardiography (biplane Simpson’s method) or by left ventricular angiography. The GRACE (Global Registry of Acute Coronary Events) score^9^ is a recognized clinical risk score that characterizes patients who present with an acute coronary syndrome. It combines age, serum creatinine, previous history of myocardial infarction, congestive heart failure, in-hospital percutaneous coronary intervention, resting heart rate, systolic blood pressure, ST segment deviation and positive cardiac enzymes. The score ranges from 1 (lowest risk) to 210 points (highest risk).

### Assessment of the Polyscore

In all participants of this study, simultaneous non-invasive 30-min recordings of the following biosignals were performed: electrocardiogram (1.6 kHz in orthogonal XYZ leads, TMS International, Enschede, Netherlands), continuous arterial blood pressure (finger photoplethysmographic device, Portapres; TNO-TPD Biomedical Instrumentation, Amsterdam, Netherlands), and respiration (piezoelectric thoracic sensor, 1.6 kHz, Pro-Tech, Porti system, TMS International). The recordings were taken during the initial hospitalisation within 2 weeks after enrolment under standardized conditions: supine resting position, quiet environment, after regular morning medication. The analysis of the recorded biosignals was performed by experienced technicians blinded to the clinical outcome data. Artifacts were eliminated and QRS-classifications were manually corrected where necessary.

As previously described^6^, the Polyscore consists of 7 parameters reflecting autonomous nervous system function that have previously been established as independent mortality risk predictors:

1. Heart rate turbulence slope (HRT slope), quantifying the RR interval deceleration following the brief acceleration after a premature ventricular contraction, was dichotomised at 2.5 ms per RR interval, as previously suggested, with values below the threshold considered pathological^10,11^.
2. Deceleration capacity (DC), quantifying the parasympathetically mediated beat to beat deceleration of the heart rate, was previously dichotomised at 2.5 ms average beat-to-beat RR-interval prolongation, with values below the threshold considered pathological^12^.
3. Baroreflex sensitivity is the quantification of the reflexive heart rate deceleration following spontaneous increases of arterial blood pressure, previously dichotomised at 1.58 ms/mmHg, with values below the threshold considered pathological^13–15^.
4. Average respiration rate, measured during the last 10 min of the recordings for optimized resting conditions, was previously dichotomised at 18.6 breaths per minute with higher values considered pathological^16^.
5. Expiration-triggered sinus arrhythmia (ETA) was dichotomised at 0.19 ms RR interval prolongation as previously suggested, with lower values indicating a pathologic reduction of respiratory sinus arrhythmia^17^.
6. Post-extrasystolic blood pressure potentiation (PESP), the ratio of the systolic blood pressure during the first post-extrasystolic beat after a PVC relative to that of the following nine sinus beats, was dichotomised at 1.03 as previously suggested, with higher values defining the presence of PESP as a predictor of mortatilty^18^.
7. The count of ≥ 7 supraventricular and/or ≥ 29 ventricular ectopic beats per 30 min was taken into account as an independent risk factor^19^ and integrated as a component of the Polyscore.

The aforementioned seven risk predictors were assessed in every study participant. According to their predefined dichotomy limits, the risk predictors were classified as normal or abnormal. The number of abnormal parameters yielded the Polyscore with 8 possible values ranging between 0 (all risk predictors normal) and 7 (all risk predictors abnormal). As predefined, patients with values ranging from 0 to 2 constituted the low-risk group, patients with values from 3 to 4 constituted the intermediate-risk group and patients with values ranging from 5 to 7 constituted the high-risk group^6^.

### Follow-up

Follow-up visits took place every 6 months for a total of 5 years. Patients who did not show up for a visit were either contacted by letter, telephone or through their general practitioner. In case a patient could not be traced, the population registry was used to identify those who died. The primary outcome was all-cause mortality and the secondary outcome was cardiac mortality over the 5-year follow-up period.

### Statistics

Continuous variables are presented as medians with inter-quartile ranges. Categorical variables are presented as absolute and relative frequencies. Hypothesis testing of differences in baseline characteristics between patients with and patients without diabetes mellitus was performed by Mann–Whitney-U test (continuous variables) or by Chi-squared test (categorical variables). A multivariable Cox proportional hazards regression model was used to simultaneously calculate hazard ratios with their 95% confidence intervals for the categorized Polyscore (using low-risk Polyscore patients as reference group) with adjustment for diabetes, age, gender, COPD, the GRACE score, and left ventricular ejection fraction. Effect modification by diabetes was assessed through inclusion of an interaction term between diabetes and the Polyscore regarding the primary endpoint. The analysis was repeated with the Polyscore as a numerical variable with values ranging from 0 to 7. The diagnostic accuracy of the model was assessed using the C-index, which is the probability that the risk estimate for a patient with an earlier event is higher than for a patient with a later event or later censoring. Kaplan–Meier survival curves together with their pointwise 95% confidence intervals were calculated for non-diabetic patients and for diabetic patients at low, intermediate and high risk according to the Polyscore. Primary and secondary endpoints for the Kaplan–Meier analysis were all-cause mortality and cardiac mortality, respectively. The Log-rank test was used to compare the Kaplan–Meier survival curves. Analyses were performed using the R 3.6.2 (The R Foundation for Statistical Computing, Vienna, Austria) and SPSS (IBM SPSS Statistics, version 26, Armonk, NY, United States). Hypothesis testing was performed at two-sided 5% significance levels.

### Ethics approval and consent to participate

The study was approved by the ethics committee of the Technical University of Munich and informed consent was obtained from all participants.

## Results

**Figure 1:**
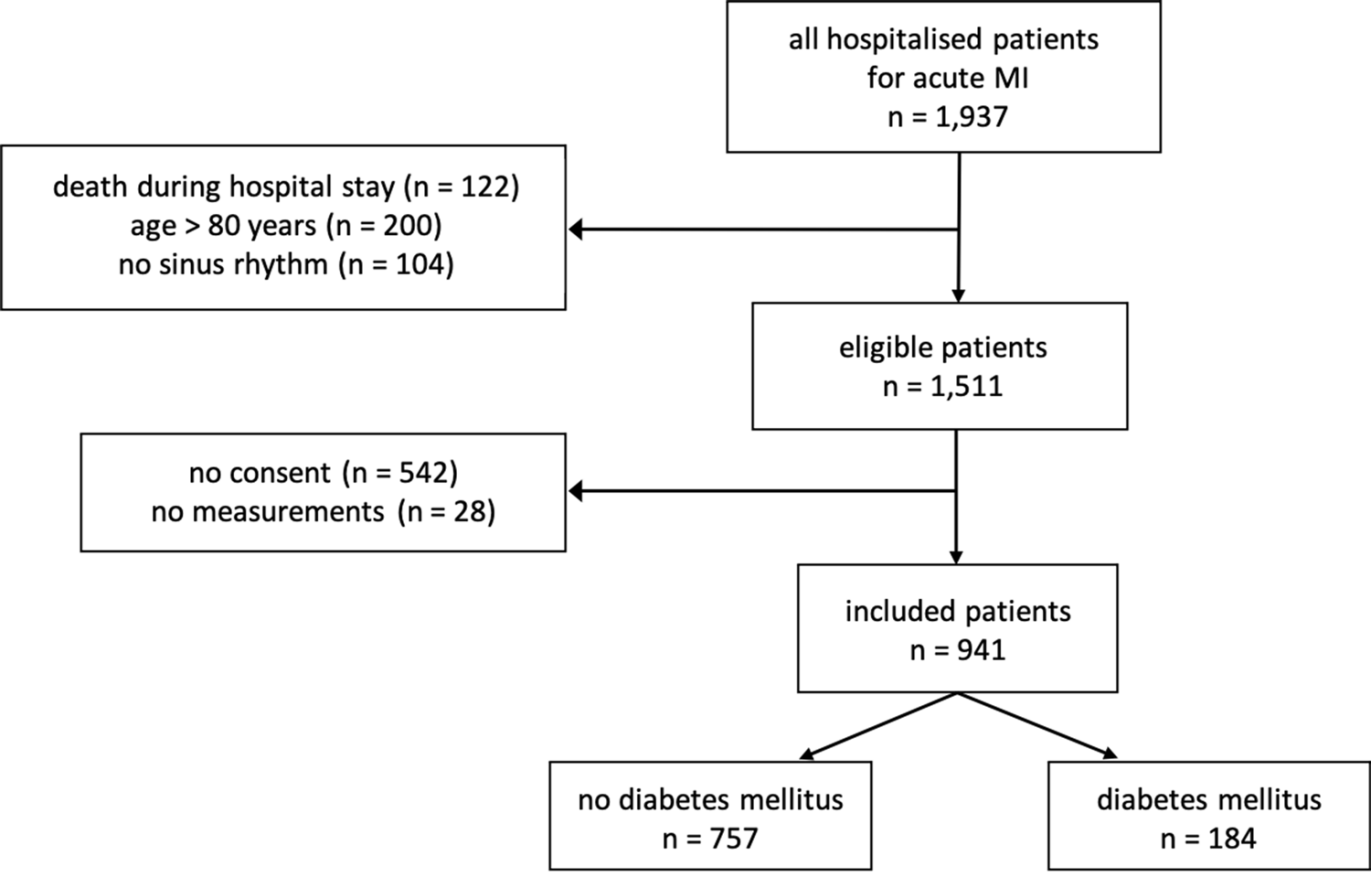
Study flow chart.

Among 1937 patients hospitalised for acute myocardial infarction in the recruiting centres, 1511 patients met the study inclusion criteria. 941 of these individuals gave written informed consent, underwent non-invasive simultaneous 30-min recordings of electrocardiogram, arterial blood pressure, and respiration, and were included for analysis. The vast majority of them (96.8%) was of European descent while the remaining were of middle-eastern or Asian descent. Of the 941 study patients, 11 (1.2%) were lost during follow-up. 184 included patients (19.6%) suffered from diabetes mellitus (Fig. 1). 51 diabetic patients (27.7%) were insulin-dependent.

Table 1 presents selected clinical baseline characteristics of the included patients with and without diabetes mellitus. Patients with diabetes mellitus were older, their body mass index was higher, they presented with a higher GRACE score, their left ventricular systolic function was lower, and they suffered more frequently from chronic obstructive pulmonary disease or arterial hypertension. No clinically relevant differences regarding acute AMI treatment or concomitant medical therapy were observed.

**Table 1:** Clinical characteristics of the study population.

	No diabetes mellitus	Diabetes mellitus	p
Number of patients, n (%)	757 (80.5%)	184 (19.6%)	
Age (years), median (IQR)	59.8 (50.7–68.0)	64.5 (57.9–71.1)	< 0.001
Female, n (%)	140 (18.5%)	42 (22.8)	0.182
BMI (kg/m^2^), median (IQR)	26.3 (24.3–28.7)	28.0 (25.4–30.8)	< 0.001
eGFR_MDRD_ (ml/min), median (IQR)	75.3 (63.8–88.3)	69.8 (58.2–82.6)	0.003
GRACE score, median (IQR)	108 (91–124)	118.3 (104–134)	< 0.001
LVEF (%), median (IQR)	53 (45–61)	50 (41–58)	0.016
COPD, n (%)	24 (3.2%)	15 (8.2%)	0.002
Arterial hypertension, n (%)	523 (69.1%)	159 (86.4%)	< 0.001
Diuretics, n (%)	99 (13.1%)	32 (17.4%)	0.015
Aspirin, n (%)	732 (96.7%)	181 (98.4%)	0.231
ACE inhibitors, n (%)	709 (93.7%)	176 (95.7%)	0.305
Betablockers, n (%)	720 (95.1%)	177 (96.2%)	0.532
PCI, n (%)	710 (93.8%)	168 (91.3%)	0.226
CABG, n (%)	5 (0.7%)	1 (0.5%)	0.858
Thrombolysis, n (%)	9 (1.2%)	5 (2.7%)	0.125
No intervention, n (%)	33 (4.4%)	10 (5.4%)	0.531

*IQR* interquartile range, *BMI* body mass index, *LVEF* left-ventricular ejection fraction, *COPD* chronic obstructive pulmonary disease, *ACE* angiotensin-converting enzyme, *PCI* percutaneous coronary intervention, *CABG* coronary artery bypass graft.

**Figure 2:**
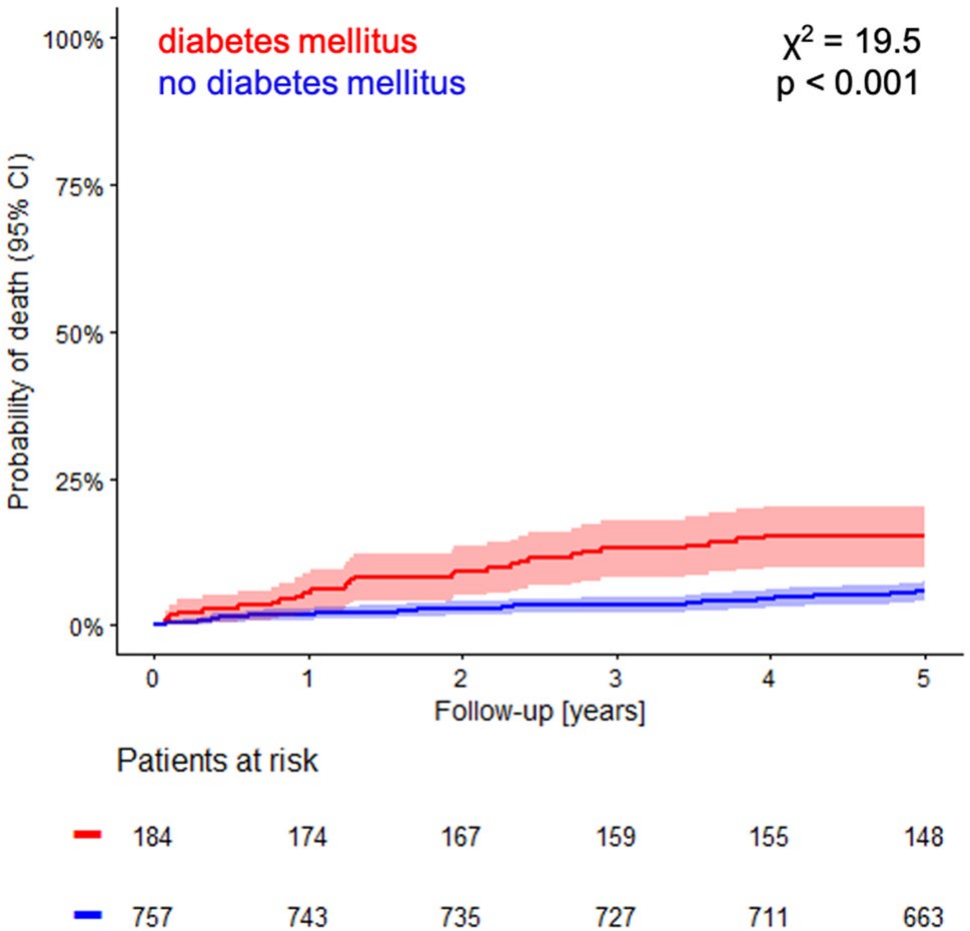
All-cause mortality in the subgroups of diabetic and non-diabetic patients. Kaplan–Meier curves of probability of death are shown. Numbers of patients at risk are listed below the time axis. *χ*^*2*^ chi-square, *95% CI* 95% confidence interval.

During the 5-year follow-up period, 72 patients (7.7%) died, with 44 deaths in the subgroup of non-diabetic patients (5.8%) compared to 28 deaths in the subgroup of diabetic patients (15.2%). The corresponding Kaplan–Meier probabilities of death are shown in Fig. 2. In non-diabetic patients 45.5% of deaths were of cardiac cause. In contrast, 53.6% of deaths in diabetic patients were cardiac deaths. Thus, the increased mortality risk of diabetic patients was even more pronounced for cardiac mortality.

**Figure 3:**
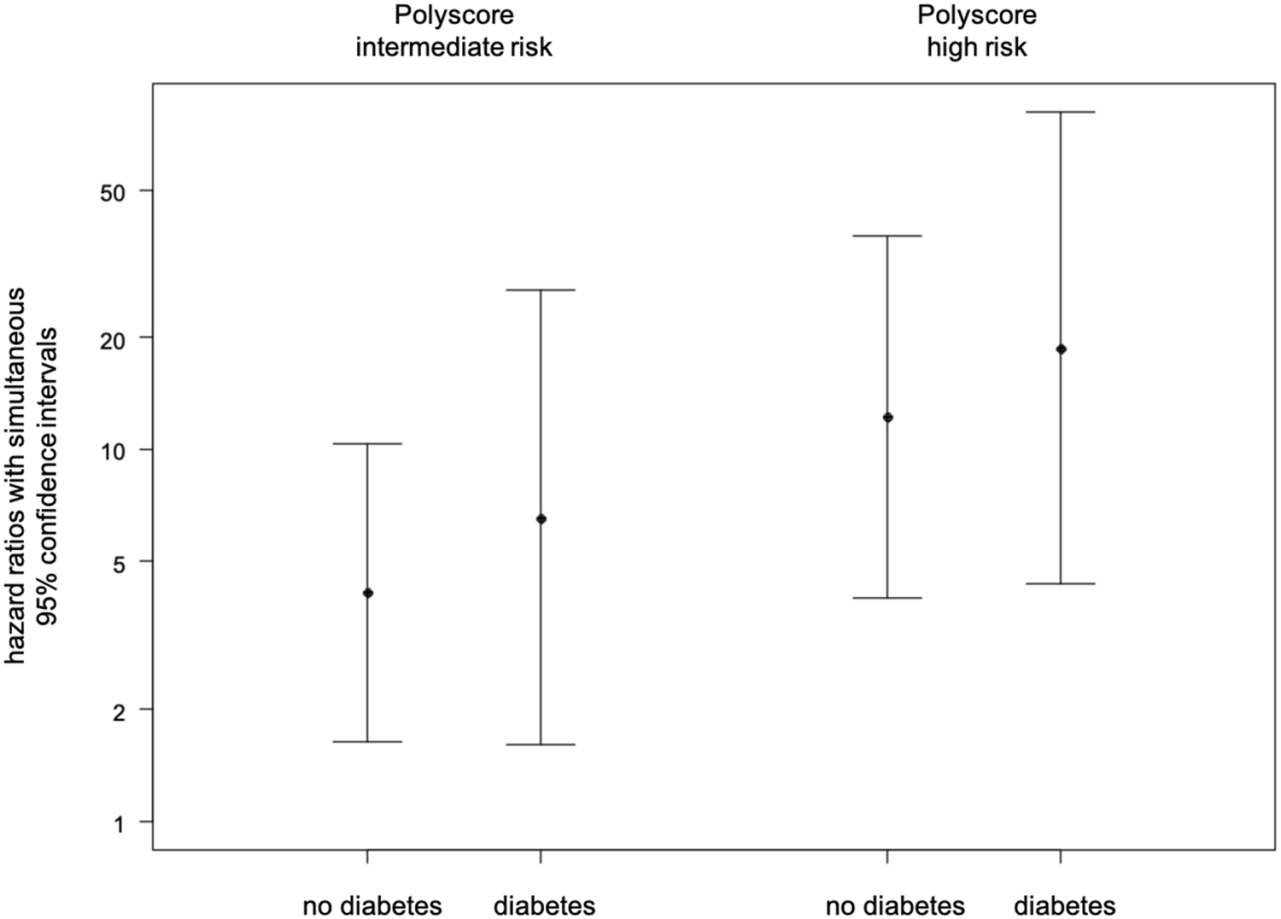
Hazard ratios with simultaneous 95% confidence intervals in patients with and without diabetes mellitus. Reference: Polyscore low risk.

In a multivariable Cox regression model including age, sex, COPD status, GRACE score, and left ventricular ejection fraction as independent covariates, the Polyscore was found to be a strong predictor of mortality both in patients without diabetes mellitus (intermediate risk: HR 4.12, 95% CI 1.64–10.37, p < 0.001; high risk: HR 12.23, 95% CI 3.99–37.52, p < 0.001) and in patients suffering from diabetes mellitus (intermediate risk: HR 6.56, 95% CI 1.61–26.8, p = 0.004; high risk: HR 18.76, 95% CI 4.35–80.98, p < 0.001) (Table 2, Fig. 3). There was no statistically significant interaction between diabetes mellitus and the Polyscore regarding mortality prediction (p for interaction = 0.775). The diagnostic accuracy of the multivariable Cox model was excellent (C-index = 0.839). The model is presented in Supplementary Table 1 for completeness. The multivariable analysis was repeated with the Polyscore as continuous variable leading to consistent results (data not shown).

**Table 2:** Hazard ratios with simultaneous 95% confidence intervals for prediction of mortality in diabetics and non-diabetics.

Polyscore	No diabetes mellitus (n = 757)			Diabetes mellitus (n = 184)		
	HR	95% CI	p	HR	95% CI	p
Intermediate risk	4.12	1.64–10.37	< 0.001	6.56	1.61–26.8	0.004
High risk	12.23	3.99–37.52	< 0.001	18.76	4.35–80.98	< 0.001

Reference: Polyscore low risk.

*HR* hazard ratio, *95% CI* simultaneous 95% confidence interval.

**Figure 4:**
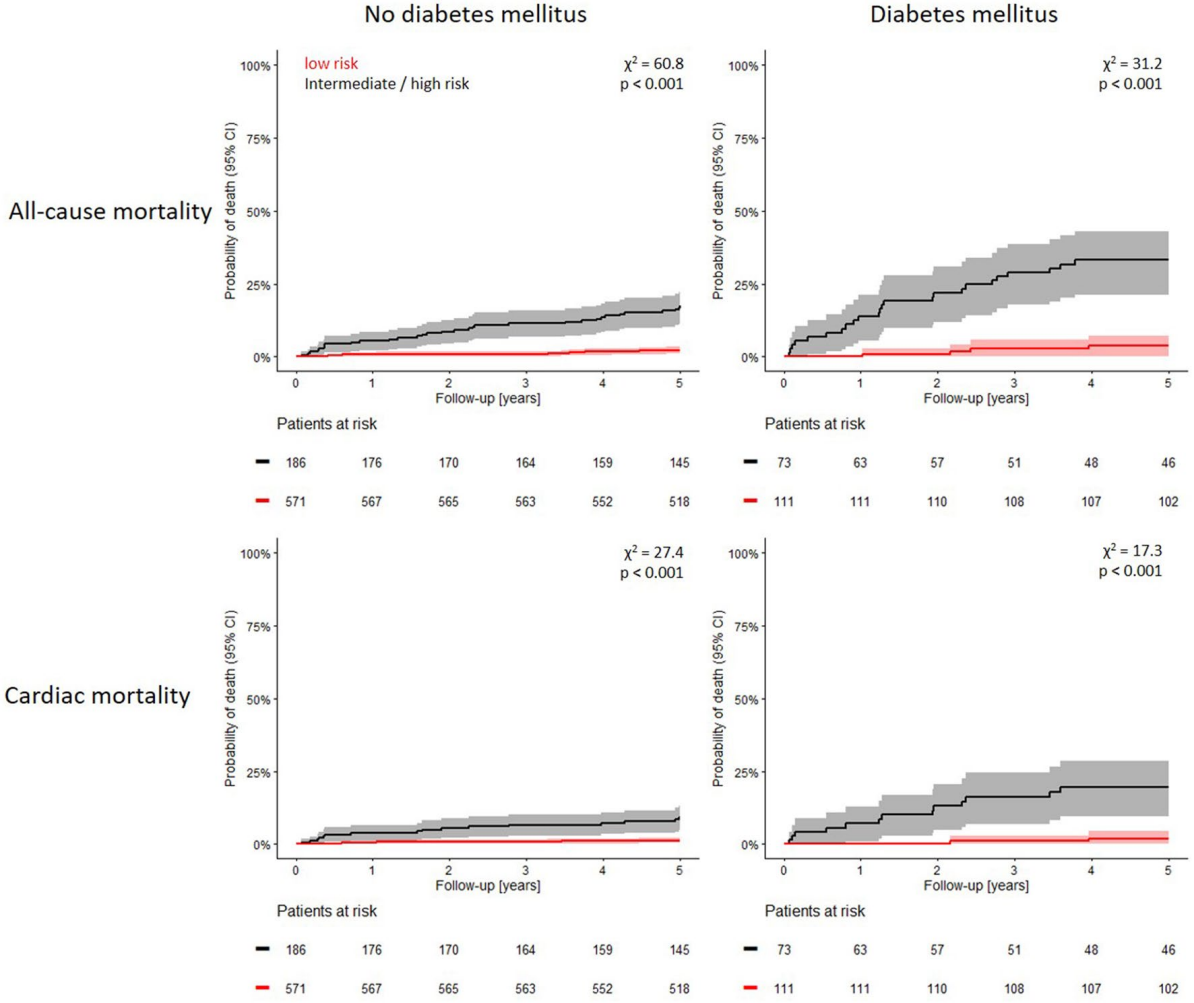
All-cause and cardiac death in patients with and without diabetes mellitus. Comparison of Kaplan–Meier probabilities of all-cause death (top panels) and of cardiac death (bottom panels) in the population subgroups defined by the Polyscore as low risk (red) and intermediate/high risk (black). The analyses were repeated separately for patients without the diagnosis of diabetes mellitus (left panels) and for patients with the diagnosis of diabetes mellitus (right panels). Numbers of patients at risk in the individual sub-groups are shown below the time axes. In all presented subsets, the differences between the probabilities of death were statistically significant. *χ*^*2*^ chi-square, *95% CI* 95% confidence interval.

The majority of both non-diabetic and diabetic patients was classified in the low mortality risk group by the Polyscore (571 [75.4%] and 111 [60.3%], respectively). The observed mortalities of these patients were low in this group regardless of diabetes status (12 [2.1%] in non-diabetic patients, 4 [3.6%] in diabetic patients) without a statistically significant difference (p = 0.339). The remaining 186 (24.6%) non-diabetic and 73 (39.7%) diabetic patients were classified in the intermediate or high-risk groups. Hence, diabetics were more likely to be classified in these subgroups of patients compared to non-diabetics (p < 0.001, Supplementary Fig. 1). The higher overall 5-year-mortality of diabetic patients was largely attributable to the subgroups classified at intermediate or high risk by the Polyscore (32 deaths [17.2%] in non-diabetic patients and 24 deaths [32.9%] in diabetic patients, p = 0.006). The differences between the cumulative probabilities of death of low-risk versus intermediate/high-risk patients were statistically significant both in non-diabetic patients (χ^2^ = 60.8, p < 0.001) and in diabetic patients (χ^2^ = 31.2, p < 0.001). Kaplan–Meier probabilities of death for diabetic and non-diabetic patients are shown in Fig. 4 (top row).

Consistent results were observed regarding the secondary endpoint cardiac mortality. In non-diabetic patients, the observed cardiac mortality was 1.2% within the low-risk group and 8.6% within the intermediate/high-risk group (χ^2^ = 27.4, p < 0.001). The observed cardiac mortality of diabetic patients within these groups was 1.8% and 17.8%, respectively (χ^2^ = 17.3, p < 0.001). Kaplan–Meier curves of cardiac mortality for diabetic and non-diabetic patients are shown in Fig. 4, bottom row.

Further Kaplan–Meier curves were obtained from diabetic patients with and without insulin therapy. In insulin-dependent patients, survival curves showed even more pronounced signals as compared with diabetic patients who did not require insulin therapy (Supplementary Fig. 2).

## Discussion

This study shows that prediction of mortality by the previously established Polyscore of autonomic risk predictors is particularly strong in diabetic survivors of myocardial infarction.

The prevalence of diabetes mellitus in the analysed cohort of post-infarction patients was 19.6%. This number is in line with the reported prevalence in comparable populations in central Europe at the time of patient enrolment^20^. As expected^21^, mortality after five years was significantly higher in individuals suffering from diabetes mellitus (15.2%) compared to non-diabetic individuals (5.8%). Accordingly, diabetic patients were more likely to be stratified by the Polyscore in the intermediate or high-risk groups compared to non-diabetics (39.7% vs. 24.6%). Furthermore, within these risk strata, mortality was substantially higher in diabetic patients (32.9% vs. 17.2%). This difference was particularly pronounced for cardiac mortality (17.8% vs. 8.6%).

On the other hand, about two thirds of all diabetic (60.3%) and non-diabetic (75.4%) patients were stratified as the low-risk group. Interestingly, the excess mortality of diabetic patients observed in the intermediate/high-risk group was much less pronounced in the low-risk group (3.6% vs. 2.1%, p = 0.339). Hence, the Polyscore is not only useful to identify non-diabetic as well as diabetic patients at risk^5,6^. In this cohort of post-infarction patients, it also identified a large subgroup of low-risk diabetic patients with a five-year mortality risk comparable to non-diabetic patients of the same subgroup. The results were consistent regarding cardiac mortality.

The Polyscore consists of seven well-established surrogate parameters of autonomic function, which may be disturbed in survivors of acute myocardial infarction and even further impaired in the presence of diabetes mellitus^7,8,22^. Therefore, it was not clear whether the Polyscore would be applicable in diabetic patients, although a previous study had shown that a combination of heart rate turbulence (HRT) and deceleration capacity (DC) predicted mortality in diabetic survivors of an acute myocardial infarction^23^. That study used 24-h Holter ECG recordings to obtain two autonomic parameters—in contrast to the Polyscore, where seven parameters (including the two of the aforementioned study) were obtained from 30-min recordings. The superior applicability of the latter approach in the clinical setting is obvious. Furthermore, the inclusion of seven different parameters may reflect autonomic function more comprehensively. Consequently, the Polyscore was highly superior in identifying patients at low risk compared to HRT and DC alone (data not shown).

The results of this study clearly demonstrate that the accuracy of the Polyscore in predicting mortality is neither impaired by the presence nor by the severity of diabetes mellitus, despite its possible impact on the autonomic nervous system. We assume that the autonomic nervous system can partly remain functional even if damaged by different pathologies. We feel that the term “autonomic reserve” adequately reflects this remaining functionality. Only in the presence of multiple different severe pathologies together, this reserve may be completely exhausted and the autonomic homeostasis lost^23^. A substantial proportion of diabetic patients at intermediate or high mortality risk according to the Polyscore may suffer from a combined autonomic failure.

The current study has important clinical implications: On the one hand, diabetic survivors of myocardial infarction are at high risk of cardiovascular events and death^21^. These patients need particular attention by the supervising physician. Close and thorough medical follow-up may prevent clinical deterioration and, in the event of deterioration, help to identify it early and respond appropriately^21,24^. Yet, current risk prediction is far from perfect from a medical and economic perspective. More effective approaches to risk stratification will be required in order to provide individualized risk-adapted and affordable medical care to this large patient group. The Polyscore may be a strong candidate for future risk prediction in cardiac patients with and without diabetes. Effective interventions for different risk groups will need to be identified. On the other hand, almost two thirds of all diabetic post-infarction patients were classified by the Polyscore in the low-risk group, with a mortality that is comparable to that of non-diabetic low-risk patients. In these patients, a more limited follow up may be sufficient to prevent complications, sparing clinical and financial resources and preventing the patients from potentially harmful therapies.

Several limitations of this study need to be considered. First of all, this study is a secondary analysis of the prospective ART (autonomic regulation trial) cohort study. External validation within an independent prospective study enrolling survivors of acute myocardial infarction with diabetes mellitus is needed. Additionally, only survivors of acute myocardial infarction aged ≤ 80 years who presented with sinus rhythm and who did not meet the criteria for primary prophylaxis by implantable cardioverter-defibrillators were investigated. Conclusions about diabetic patients with other conditions cannot be drawn from these results. These questions need to be addressed by further investigations. In our study, we used all-cause mortality as primary endpoint and cardiac mortality as secondary endpoint. Other outcomes such as reinfarction, stroke, etc., may be considered in future studies. Due to the lack of information about the autonomic function before the index myocardial infarction, it is not possible to attribute potential autonomic dysfunction to the recent myocardial infarction or to the preexisting diabetes mellitus. Another limitation is the lack of information about the glycemic status including the levels of advanced glycation end products, the details of the prescribed antidiabetic therapies, and the duration of diabetes at the time of study enrolment. Moreover, standardized reflex assessment as suggested by Ewing et al. was not performed^25^.

## Conclusions

The Polyscore is a strong and independent mortality risk predictor in survivors of an acute myocardial infarction who simultaneously suffer from diabetes mellitus. It stratifies almost two thirds of diabetic patients at low risk of death, comparable to the risk of non-diabetic low-risk patients. A comprehensive risk-adapted follow-up strategy based on the Polyscore with focus on the much smaller groups of patients at intermediate and high risk may render patient care more efficient and cost-effective.

## Supplementary Information

Supplementary Information.

## Data Availability

The datasets used and/or analysed during the current study are available from the corresponding author on reasonable request.

## Abbreviations

95% CI: 95% Confidence interval
χ^2^: Chi-square
ACE: Angiotensin-converting enzyme
ART: Autonomic regulation trial
BMI: Body mass index
CABG: Coronary artery bypass graft
COPD: Chronic obstructive pulmonary disease
ECG: Electrocardiogram
HR: Hazard ratio
GRACE: Global Registry of Acute Coronary Events
IQR: Interquartile range
LVEF: Left-ventricular ejection fraction
PCI: Percutaneous coronary intervention
