# Supplementary material for: The Polyscore of autonomic parameters predicts mortality and identifies low-risk individuals among diabetic survivors of acute myocardial infarction
Source: Sci Rep. 2022 Apr 12;12:6069. doi: 10.1038/s41598-022-09899-y (PMC9005709; doi:10.1038/s41598-022-09899-y)

Supplementary Information

The Polyscore of autonomic parameters predicts mortality and identifies low-risk individuals among diabetic survivors of acute myocardial infarction

Alexander Steger, Michael Dommasch, Alexander Müller, Petra Barthel, Daniel Sinnecker, Larissa Wieg, Alexander Hapfelmeier, Helene Hildegard Heidegger, Katharina Maria Huster, Eimo Martens, Karl-Ludwig Laugwitz, Georg Schmidt, Ralf Dirschinger.

Supplementary table 1 Multivariable Cox regression model for the prediction of mortality

Variables	hazard ratio (95%-confidence interval)	regression coefficient ± standard error	p
age (years)	1.01 (0.96 – 1.05)	0.01 ± 0.02	0.796
Sex (m / f)	0.82 (0.45 – 1.48)	-0.20 ± 0.30	0.499
LVEF (%)	0.99 (0.97 – 1.01)	-0.01 ± 0.01	0.259
COPD (yes / no)	2.34 (1.18 – 4.67)	0.85 ± 0.35	0.015
GRACE score	1.02 (1.01 – 1.04)	0.02 ± 0.01	0.007
diabetes (yes / no)	1.17 (0.37 – 3.70)	0.16 ± 0.59	0.787
Polyscore intermediate risk	4.12 (1.97 – 8.62)	1.42 ± 0.38	< 0.001
Polyscore high risk	12.23 (4.99 – 29.96)	2.50 ± 0.46	< 0.001
Diabetes (yes/no) x Polyscore = intermediate risk	1.59 (0.42 – 6.09)	0.47 ± 0.69	0.498
Diabetes (yes/no) x Polyscore = high risk	1.53 (0.37 – 6.36)	0.43 ± 0.73	0.555

LVEF, left-ventricular ejection fraction; COPD, chronic obstructive pulmonary disease; x, interaction term of named variables.

Supplementary figure 1 Distribution of Polyscore risk strata in patients with and without diabetes mellitus

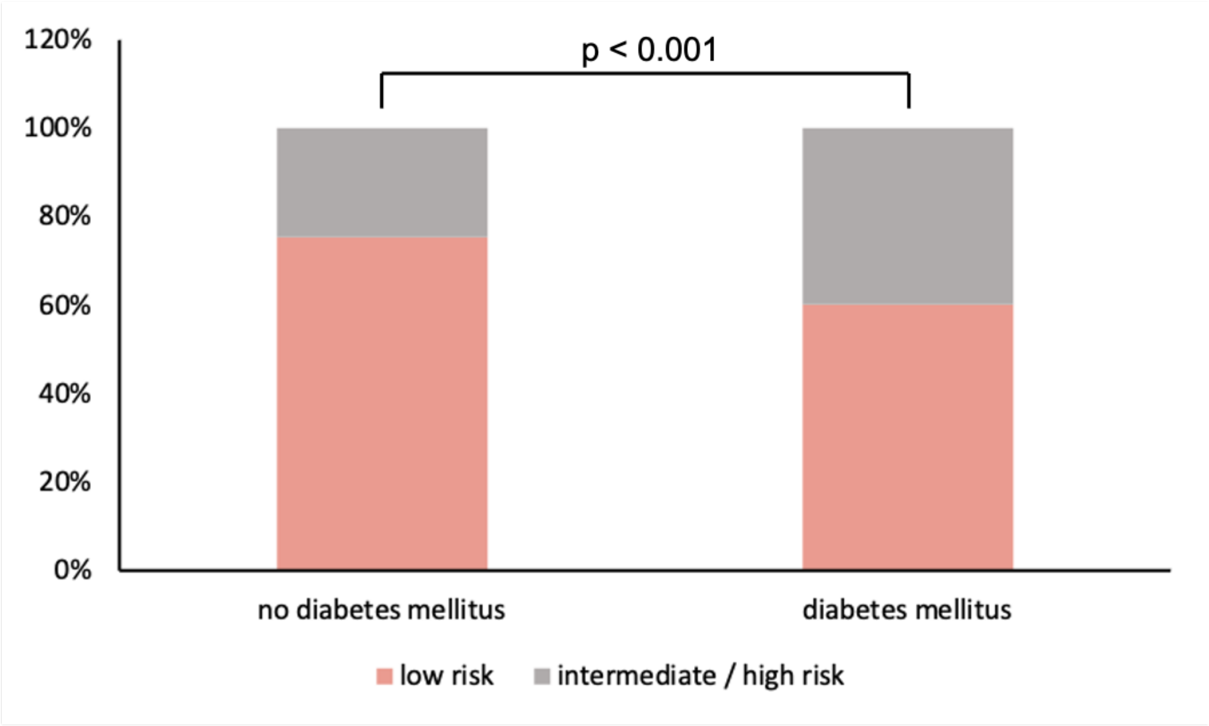

Supplementary Figure 2 All-cause and cardiac death in diabetic patients with and without insulin therapy

Kaplan–Meier probabilities of all-cause death (top panels) and of cardiac death (bottom panels) in the population subgroups defined by the Polyscore as low risk (red) and intermediate/high risk (black). The analyses were repeated separately for diabetic patients without insulin therapy (left panels) and for diabetic patients with insulin therapy (right panels). Numbers of patients at risk in the individual sub-groups are shown below the time axes. In all presented subsets, the differences between the probabilities of death were statistically significant

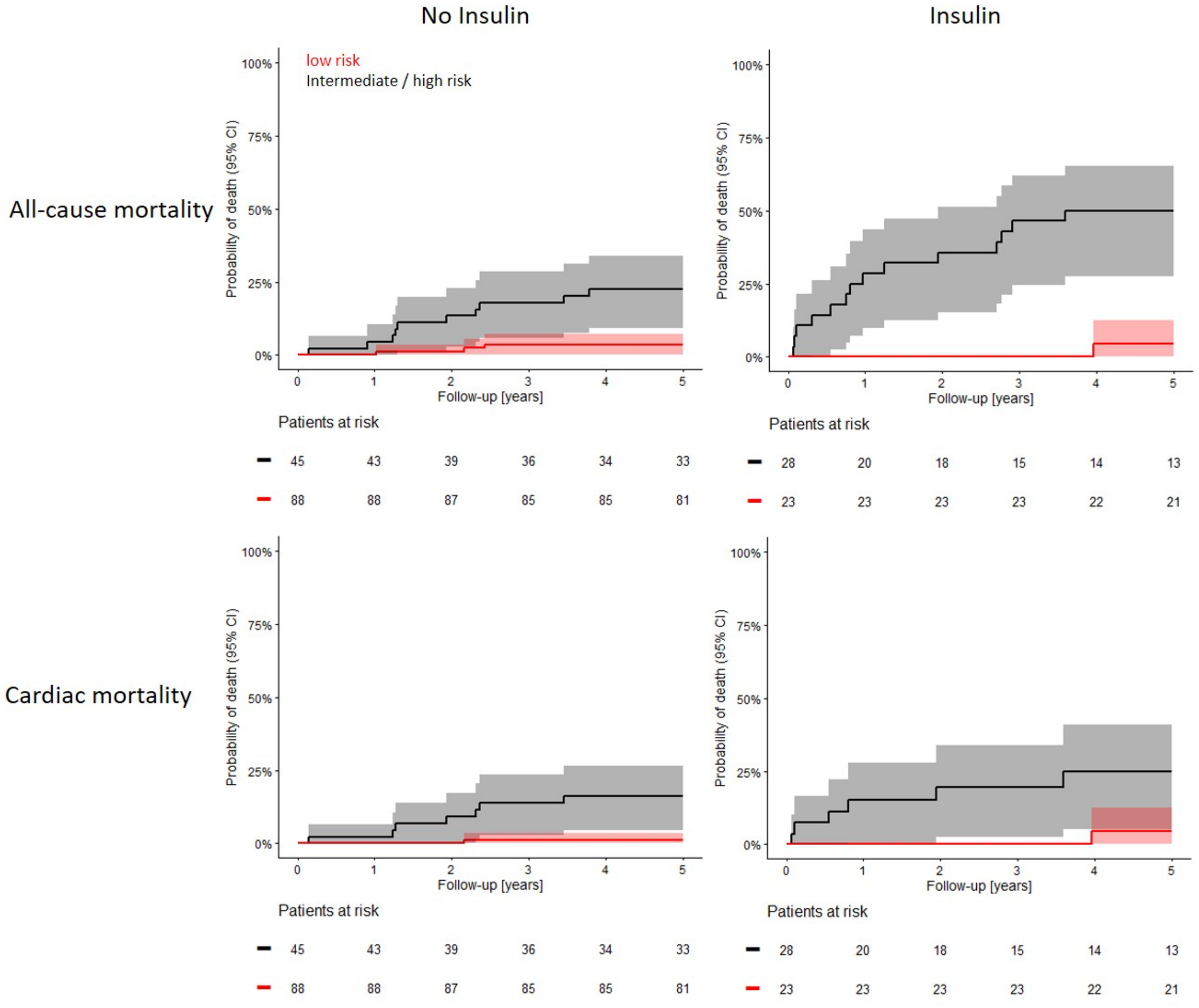

Supplement: Supplementary file 1 — Supplementary Information. [file 41598_2022_9899_MOESM1_ESM.pdf]
